# Sub-Dural Haematoma After Accidental Dural Puncture During Labour Epidural Analgesia

**DOI:** 10.7759/cureus.16915

**Published:** 2021-08-05

**Authors:** Gisha V Mathew, Khalil Shibli, Noureddine Korichi, Venkatesh B Thippeswamy

**Affiliations:** 1 Anaesthesia, Hamad Medical Corporation, Doha, QAT

**Keywords:** subdural hematomas, post dural puncture headache, labour analgesia, dural puncture, combined spinal epidural

## Abstract

Inadvertent dural puncture with subsequent post-dural puncture headache (PDPH) is the most typical complication of labour epidural analgesia. Subdural hematoma (SDH) is a rare but late neurological complication of this procedure. The intracranial hypotension created by the cerebrospinal fluid (CSF) leakage through the dural defect can lead to the rupture of the bridging veins to produce a subdural hematoma. A change in the character from postural to a non-postural headache is a warning sign of subdural hematoma. We describe a case of post-dural puncture headache followed by the development of cranial SDH in a patient who refused a blood patch and opted for conservative treatment. We conclude that a high index of suspicion must be maintained with a witnessed dural puncture or even without it but having persistent headache, to detect any severe complications like an intracranial subdural hematoma. An epidural blood patch is to be considered when the headache does not subside with conservative management. Early employment of MRI or CT head imaging studies should be considered to exclude, diagnose, or treat any serious complication without unnecessary delay.

## Introduction

Epidural analgesia is considered the standard of care in Obstetric anaesthesia practice. However, the most typical complication is inadvertent dural puncture with consequent post-dural puncture headache (PDPH). PDPH may typically begin 24 to 48 hours after an accidental dural puncture. We describe a case of post-dural puncture headache followed by the development of cranial subdural haematoma (SDH) in a patient who refused a blood patch, opted for conservative treatment and got herself discharged from the hospital despite the unresolved headache. Later, the patient developed cranial SDH and underwent burr hole surgery under general anaesthesia with full neurological recovery.

## Case presentation

This is a case of a 23-year-old gravida 2, para 1 patient with a history of one previous uneventful lower segment cesarian section (LSCS) under sub-arachnoid block, only 12 months ago. At this visit, vaginal delivery was planned and attempted. Platelets and coagulation parameters were within the normal range. An on-call anaesthetist was called into the labour room to place an epidural in this 48 kg patient. The anaesthetist noticed a deviation of the spine, which was later confirmed by the MRI as mid-lumbar scoliosis to the right at the L3-4, L4-5 region, about 3 cm from the midline. She had not reported any previous history of cerebral trauma, vascular malformations, or neurological disorders. Under all aseptic precautions, the epidural was sited in the sitting position with 2 ml of 2% lidocaine local infiltration. Since the patient had frequent contractions, she moved during the procedure, and the epidural needle was removed once. In the second attempt, the epidural space reached at 4 cm with a 16 G Touhy needle by loss of resistance to saline (LORS) technique. The procedure was uneventful, and there was no witnessed dural puncture with the Tuohy needle. A combined spinal-epidural (CSE) was performed using a 27 G Whitacre needle by the needle through needle technique. Having obtained clear cerebrospinal fluid (CSF), 2.5 mg of heavy bupivacaine with fentanyl 25 μg was injected intrathecally. Subsequently, the epidural catheter was introduced and left 5 cm in situ and secured at 9 cm at the skin. After confirming a negative aspiration, an infusion of 0.1% levobupivacaine with 2 μg fentanyl/ml at the rate of 10 ml per hour was commenced through a patient-controlled epidural analgesia (PCEA) infusion pump, with 5 ml boluses on demand every 30 minutes. 

Sensory level to cold sensation was checked after one hour using ethyl chloride spray and found to be at T5, and the patient was unable to move her legs. After stoppage of PCEA infusion, clear fluid could be aspirated immediately through the epidural catheter. After 30 minutes of stopping the infusion, sensory and motor levels were rechecked during which time she could move her legs, and the sensory level had regressed to T10-T11. She could now feel mild pain of the contractions. Therefore, infusion was restarted at a lower rate of 3 ml/hour with boluses of 4 ml every 30 minutes on demand, the setting at which the patient was comfortable.

Nine hours into labour, the patient had a painless delivery of a female baby weighing 3.025 Kg with an APGAR score of 9 and 10 at 5 and 10 minutes. The epidural catheter was removed after one hour of delivery. The patient was followed the next day and had a mild fever (38.2 degrees); therefore she was started on ceftriaxone.

On day two (48 hours) patient developed a mild headache and was reassured by the surgeons. However, the headache worsened on day three (72 hours) and was seen by the anaesthetist. Upon examination, the symptoms were suggestive of a PDPH, and the patient was started on conservative management, including increased oral fluids, NSAIDs, caffeine and confinement to bed. The patient was advised to stay in the hospital for observation, but she refused and got discharged. She was advised to come back in case of worsening headache or new symptoms.

The patient returned to the ED on day five with a worsening headache and was seen by the obstetrician and the consultant anaesthetist. She was admitted by the on-call anaesthetist for two to three days for the management of the headache and was offered an epidural blood patch which she declined. Hence she was conservatively managed all the while re-counselling her for a blood patch.

**Figure 1:**
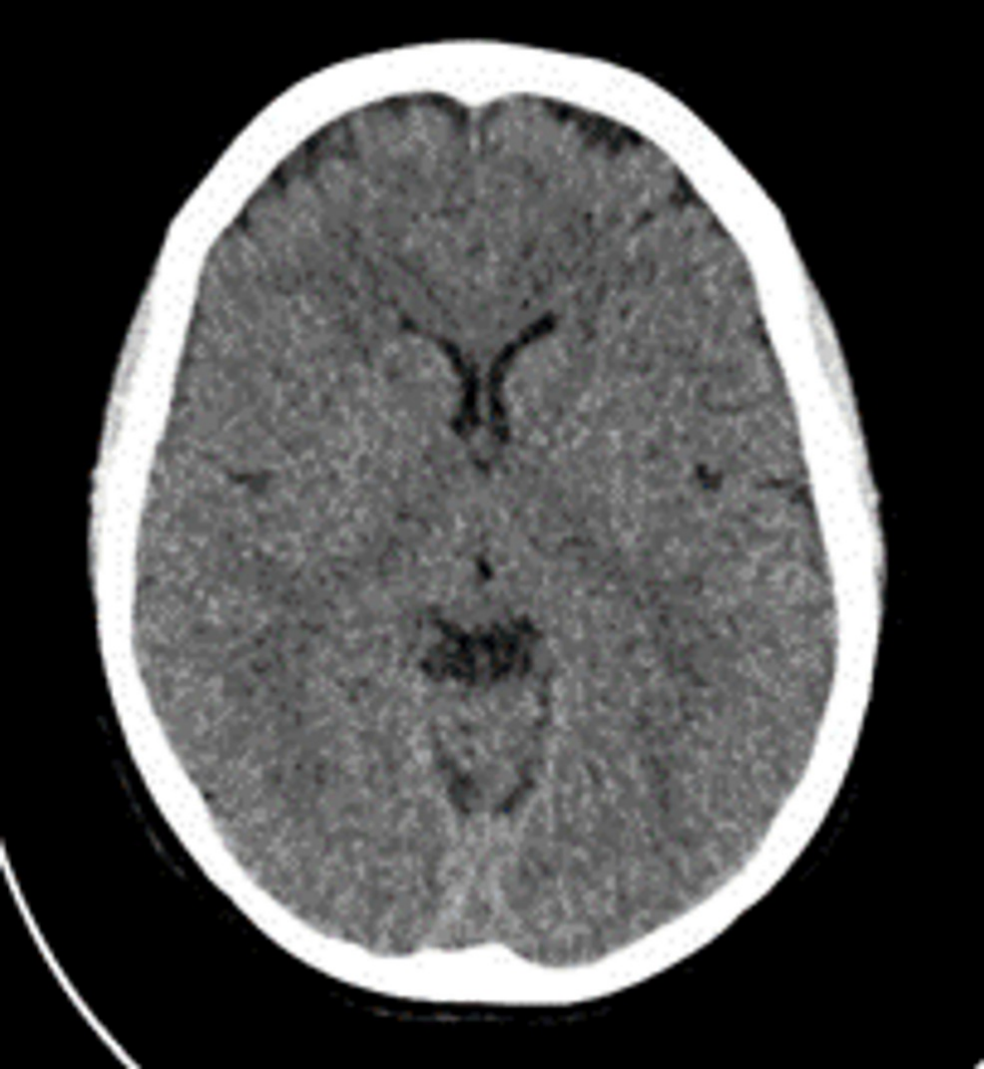
CT Head day 7

On day six and day seven, the headache persisted and, an urgent CT scan of the brain was done (Figure 1), and the neurologist consulted. In the CT Brain, no acute intracranial abnormalities were detected at the time. The patient was considered for an epidural blood patch, to be done on the following morning (day eight) but not undertaken since the patient reported no headache and seemed unwilling for the blood patch. The headache started again on day nine, so the anaesthetist again suggested the blood patch. Still, the patient refused and got herself discharged the following day, with the advice to continue the analgesics for three days.

**Figure 2:**
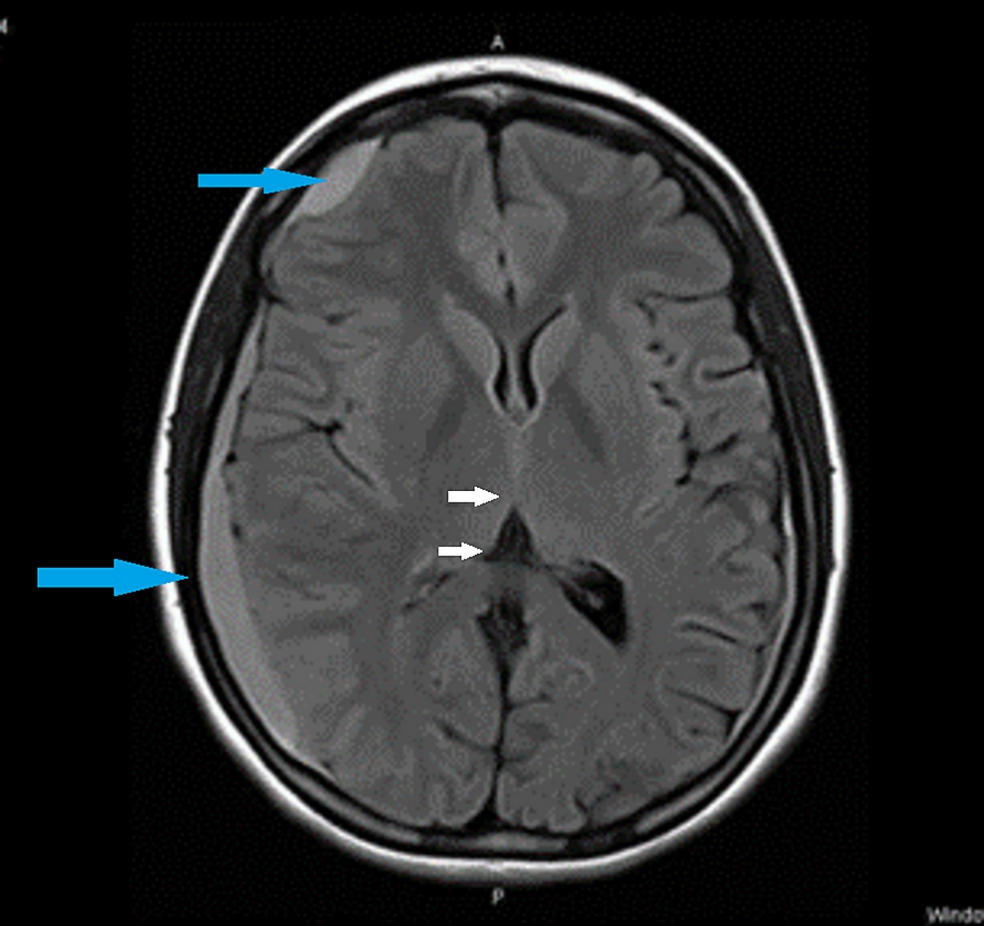
MRI day 25

Four days after self-discharge, she returned to the ED on day 14 with a severe headache and numbness on the left half of the face, left hand, and left leg. She was immediately referred to the neurologist, but she chose to go home only to come back again with a headache on day 15. The ED physician wanted to rule out a transient ischemic attack or migraine, and a routine neurology clinic appointment was obtained after 10 days. On day 25, the neurologist provisionally diagnosed the headache as migraine and ordered an MRI/MRA Brain (Figure 2) which revealed a right fronto-parieto-occipital ageing SDH, measuring 16.1 mm in size (large blue arrows), causing a midline shift of 5.5 mm (small white arrows).

**Figure 3:**
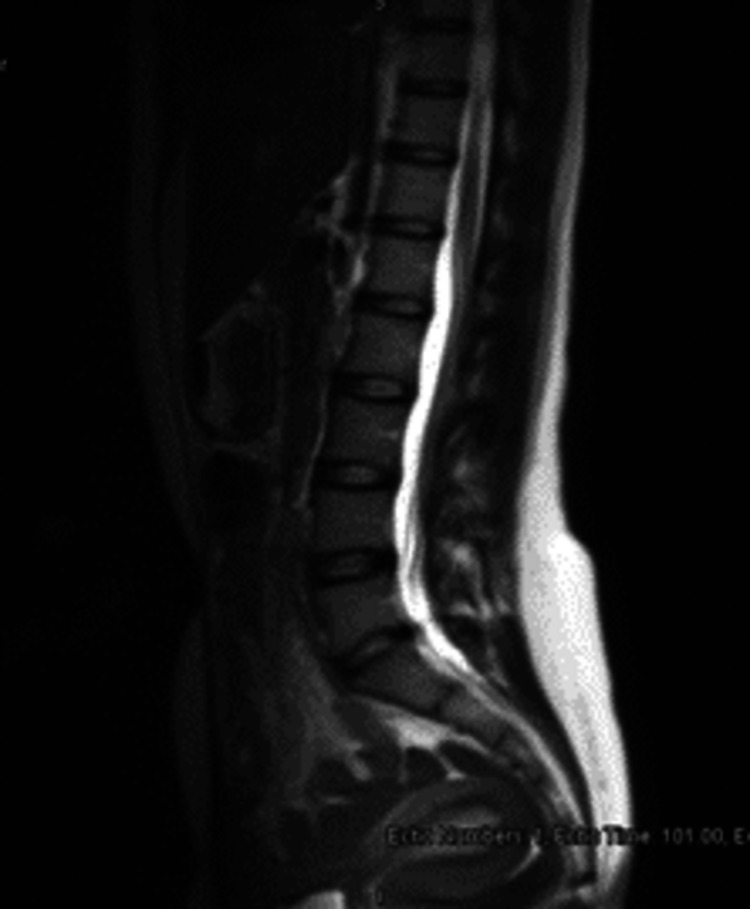
MRI Spine day 31

Neurosurgery consultation was sought on this MRI, and since the patient was asymptomatic with no focal neurological deficits, and a Glasgow Coma Score (GCS) of 15/15, the neurosurgeons opted for conservative management and advised to visit the OPD four weeks later or to report in the ER in case of any new signs or symptoms. On day 31, the patient came back to the ER complaining of a headache on and off, and an MRI Spine was done to look for any CSF collection. This MRI spine (Figure 3) showed hypertrophied ligamentum flavum and mid-lumbar scoliosis with no extradural CSF collection.

**Figure 4:**
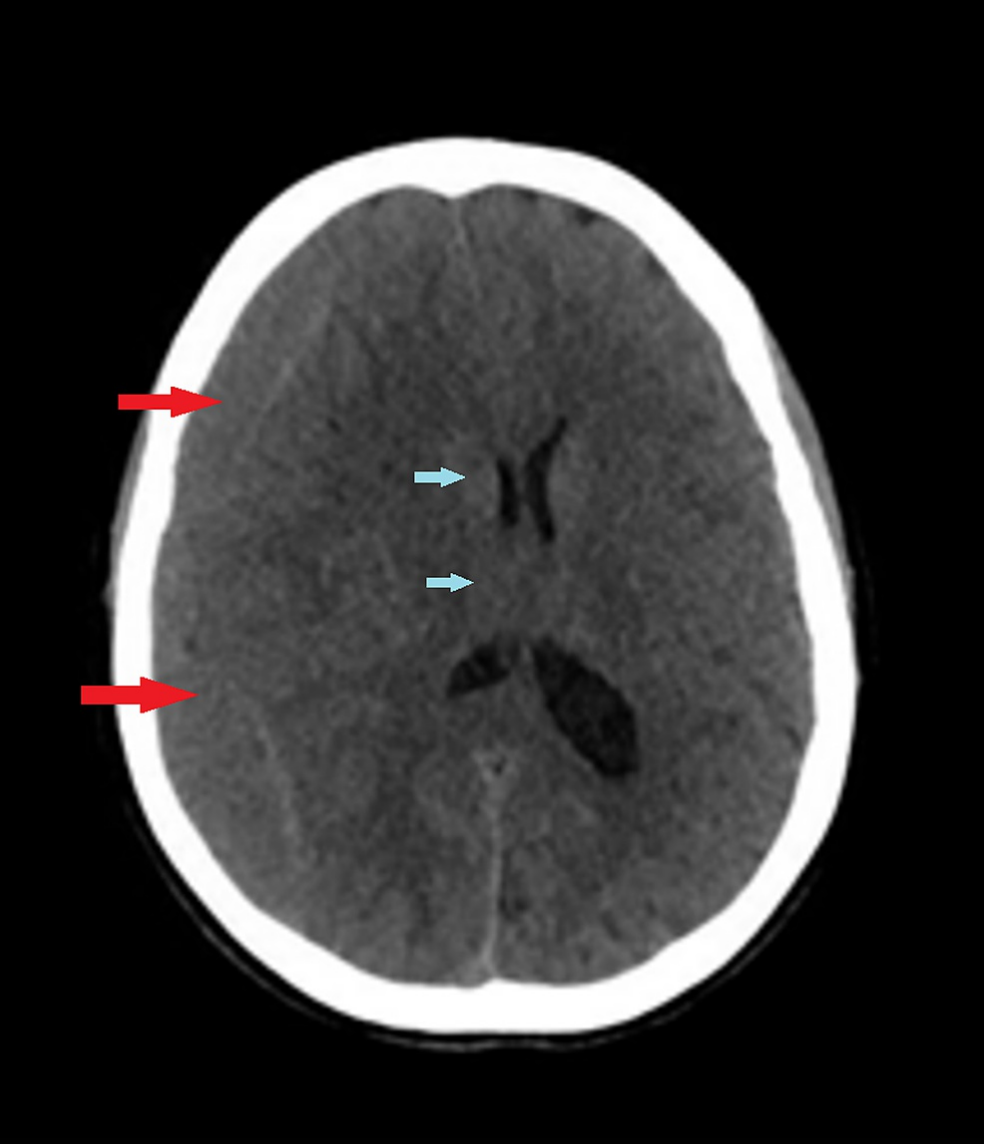
CT Head day 48

On Day 48, she revisited the ER with a worsening headache for the past three days. A CT brain was repeated. The cranial CT scan (Figure 4) showed a subacute SDH in the right fronto-parietal region with an increase in haematoma size to 25 mm thick (large red arrows) and a midline shift of 9 mm (small blue arrows).

**Figure 5:**
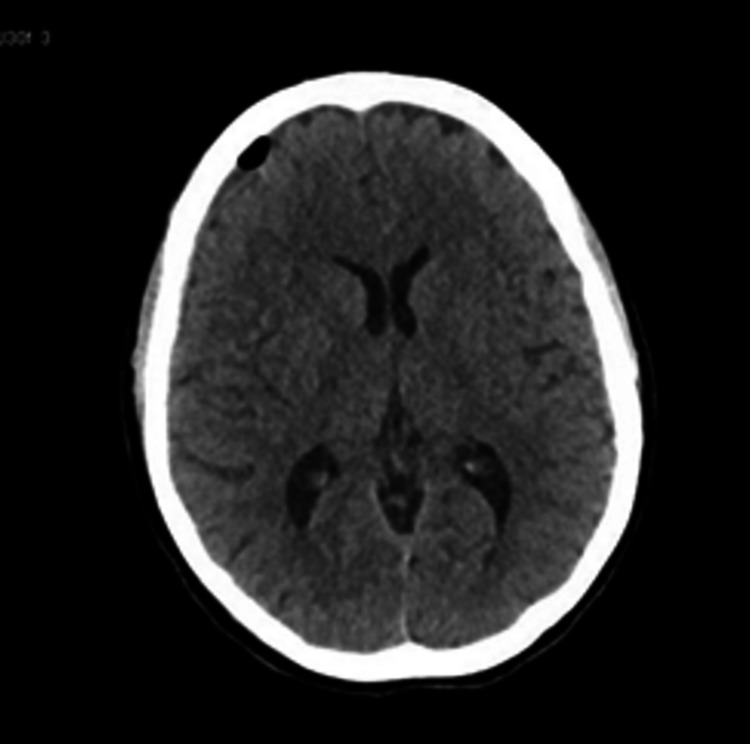
CT Head day 51

The patient was immediately admitted for burr hole evacuation of the SDH and was operated on the next day. The hematoma was evacuated through two burr holes. On the third postoperative day (day 50), the cranial CT showed a near-total evacuation of the right frontoparietal SDH without any midline shift. On day 51, CT Head (Figure 5) repeated and showed a re-expansion of the right subarachnoid space and dried lateral ventricles. She was discharged two days later (day 53) and had recovered completely with no focal neurological deficits.

## Discussion

In obstetric anaesthesia, the incidence of PDPH after an inadvertent dural puncture with a Tuohy needle is almost up to 80% [1]. The untreated PDPH after discharge could be lethal and near detrimental, as it was in this case. The only exception is that the patient was followed up diligently, but she opted out of hospital care. The clinical diagnosis of PDPH was made immediately, and the patient was directed for further investigations, which she declined. While PDPH is a benign self-limiting condition, post-dural puncture sub-dural haematoma (PDPSDH) is a rare, potentially fatal complication [2]. The intracranial hypotension created by the CSF leakage through the dural defect can lead to the rupture of the bridging veins to produce a subdural hematoma (SDH). In SDH, it is typically a non-postural headache associated with vomiting, convulsions, and other neurological symptoms. A change in the character from postural to a non-postural headache is a warning sign of subdural hematoma [1]. Other causes like preeclampsia, migraine, meningitis and cerebral venous thrombosis should also be considered in a non-postural headache. A computed tomography (CT) scan is the most effective and easiest way to detect SDH. Hematomas of less than 5 mm usually resolve spontaneously and need only close observation [3]. A CT scan of the brain gives a correct diagnosis; however, an intracranial hematoma of more than seven to 21 days old cannot be distinguished from the brain tissue. An MRI or CT with contrast may be needed to confirm the diagnosis. Surgical management should be considered in any patients with CT or MRI evidence of significant haematoma, neurological impairment or symptomatic brain compression.

Delayed development of SDH was also reported in a case of a 32-year-old who underwent elective cesarean section under spinal anaesthesia. She developed headaches and nausea 10 days postoperatively, which progressively worsened. On the 40th postoperative day, the patient was admitted to the emergency room with hemiparesis on the left side of the body. Urgent Brain MRI revealed a subacute subdural hematoma, with progressive neurologic signs and mass effect and midline cerebral shift symptoms. However, the patient received surgical decompression with complete recovery. Authors concluded that a high index of suspicion for the pattern and characteristics of headache with a meticulous neurological examination could help diagnose such serious events timely to be treated adequately [4]. A similar case was reported about a 25-year-old woman, recipient of epidural analgesia for labour pain with subsequent PDPH, who had received a blood patch with no improvement in symptoms. Brain imaging showed a bilateral subdural hematoma. The patient was treated with steroids and non-surgical management. Authors concluded that PDPH with a lack of response to established medical treatment should raise suspicion for a cerebral event. Head imaging must be performed to exclude or delineate cranial hematoma [5].

Grace et al. reported delayed appearance of SDH in eleven obstetric patients after labour epidurals over seven years at a tertiary care hospital. In 10 of the 11 cases, SDH was diagnosed within one to seven days after the performance of labour epidural analgesia except for one case, which was diagnosed on the 25th day. Although 10 of 11 (91%) patients had a second hospital stay for two to four days for observation, they did not require any neurosurgical intervention. Out of 11, only one case (9%) had decompressive hemicraniectomy after becoming unresponsive. The authors reported the observed rate of labour epidural analgesia associated with SDH as 0.026% (11 in 42,969, about 1:3900). The rate of SDH was 1.1% (five in 437, about 1:87) if a recognized dural puncture occurred during epidural catheter placement [6].

The unintentional dural puncture and SDH may be complicated if coagulopathy develops after the placement of epidural catheter, as reported in a patient who developed postpartum bilateral intracranial subdural hematoma with hemolysis, elevated liver enzymes and low platelets (99,000/µL). The patient developed a headache on day one, but an epidural blood patch (EBP) could not be performed due to a low platelet count of 21,000/µL. The headache soon became non-postural, and MRI revealed bilateral temporal subdural hematomas. The patient was managed conservatively and discharged home with no residual neurological deficit. The authors concluded that thrombocytopenia together with possible abnormal platelet function increased the risk of subdural hematoma. Early resort to look for alternative diagnoses to PDPH should be considered whenever headache is not posture related [7].

A large recent cohort study of 22,130,815 deliveries looked at the association of PDPH and incidence of SDH found that there was a PDPH rate of 309 per 100,000 deliveries and an SDH rate of 1.5 per 100,000 deliveries. For women with post-dural puncture headaches, the SDH rate increased significantly, to 147 per 100,000 deliveries. This study concluded that the women with post-dural puncture headaches after neuraxial anaesthesia during childbirth had an increased risk of being diagnosed with intracranial subdural hematoma [8].

Srivastava et al. report a case of intracranial SDH, which developed 11 days after spinal anaesthesia for caesarean section. The patient complained of headache on the second postoperative day, relieved by analgesics, bed rest, and hydration. However, subsequently, she presented with severe headache, vomiting, dizziness, dysarthria, irritability, and somnolence culminating in the diagnosis of left-sided SDH, which was confirmed radiologically and treated surgically with complete patient recovery. This report highlights the need of considering the possibility of SDH in patients when post-dural puncture headache is prolonged or recurs after a headache-free period with neurological symptoms [9].

Intracranial subdural hematoma due to the puncture of spinal dura mater is extremely rare, with less than 100 cases reported, and often initially misdiagnosed and treated simply as a PDPH. For example, in a simple four-day post-epidural procedure headache, a patient was diagnosed with bilateral intracranial subdural hematomas on CT. Repeat imaging and careful clinical examination and follow up revealed spontaneous hematoma resolution, and no surgical intervention was required. Szeto et al. concluded that the headaches are common in the postpartum period, often after receiving epidural or spinal anaesthesia, and intracranial subdural hematoma may occur as a rare complication. However, the authors suggest keeping a high index of suspicion when evaluating patients with post-spinal or epidural procedural headaches [10].

An interesting case of a 41-year-old patient with an unruptured cerebral aneurysm was reported with an accidental dural puncture during CSE for elective LSCS, where no headache was reported. Since CSF was identified during puncture of the epidural space, and a separate catheter was sited after re-puncture, a high index of suspicion was maintained. However, the patient had no headache but had slight neck stiffness on the second postoperative day. MRI on the fourth postoperative day showed a bilateral subdural hematoma that showed an increase in size on CT done on the ninth postoperative day. An epidural blood patch was performed because the patient had a witnessed CSF leak at the siting of the epidural catheter. Subsequently, a decrease in the size of the hematoma was confirmed on the 11th postoperative day by CT imaging, and the patient was discharged. The patient had not developed any additional symptoms. In this case, the authors suggest that a witnessed dural puncture with other allied symptoms, even in the absence of headache, may detect some seriously developing complications like a subdural hematoma in brain imaging studies [11].

## Conclusions

We conclude that a high index of suspicion must be maintained with a witnessed dural puncture or even without it but having headache, to detect any severe complications like intracranial subdural hematoma, if a patient does not feel right or complain of nausea, vomiting, neck pain and dizziness. An epidural blood patch is to be considered when the headache does not subside with conservative management. All the case reports and case series reported early employment of MRI or CT Head imaging studies to exclude, diagnose, or treat any serious complication without unnecessary delay.
